# Supplementary material for: Patterns of Intron Gain and Loss in Fungi
Source: PLoS Biol. 2004 Nov 30;2(12):e422. doi: 10.1371/journal.pbio.0020422 (PMC532390; doi:10.1371/journal.pbio.0020422)
Supplement: Table S1 — Also available at http://genes.mit.edu/NielsenEtAl/. (4.3 MB ZIP). [file pbio.0020422.st001.zip › NielsenEtAl/html/413.html]

AN1534.1.NCU00502.1.MG04752.1.FG00373.1


```
 CLUSTAL W (1.82) Multiple Sequence Alignments - Introns Inserted


Sequence 1: NCU00502.1	241 aa
Sequence 2: MG04752.1	257 aa
Sequence 3: FG00373.1	242 aa
Sequence 4: AN1534.1	242 aa
Alignment Length: 257 aa
Number Identitical Residues: 140 aa
Alignment Score (without introns) 6267


MG04752.1 	MASRLARTALV1LLTKVSSLFAG~AARTRPSTAFAQRTALPALTSLTTARASSNVPTQDP
NCU00502.1	MASRLARTA--~---------VG1AARLRPS---VVPRVLPALSTVASPRYSSGVPSEDP
FG00373.1 	MASRLARSA--~---------VG1APLLRPV---LARRAAPAFS-VAAARYNSNVPAEDP
AN1534.1  	MASRLAKSA--~---------IG1VTRLRPT---LASRNVAAVT-LSATRAASNVPTEDP
          	******::*             * ..  **          .*.: :::.*  *.**::**

MG04752.1 	KTKAQSLIDSLPGSSLVSKTAILSSAAGLSIFAISNEFYVVNEETVVAFCLLSVWGALIK
NCU00502.1	KTKAQSIIDSLPGSNLMSKTAILSSAAGLSIYALSNEYYVVNEETVVAFCLLSVWGGLIK
FG00373.1 	KKKAQSIIDALPGNSLLSKSAILSSAAALSVYAISSEYYVMNEETIIAISLLSVWGALIK
AN1534.1  	KQKAQSILDSLPGNSLVSKTAILSATAGLSIAAISNELYVMNEETVAAFCLLSVFTAVAK
          	* ****::*:***..*:**:****::*.**: *:*.* **:****: *:.****: .: *

MG04752.1 	FGGPMYSQWADSQSNKIKDILNAARADHTQAVKNRIENVQQMSGVIDITKSLFAVSK0ET
NCU00502.1	FGGPLYKKWADEQSDKIKNILNSARADHTQAVKTRIGDVKQMSGVIDITKTLFAVSK0ET
FG00373.1 	YGGPAYKEWAEAQNNKIKNILNSARADHTEAVKGRIEDVKQMGSVVEITKNLFQVSK0ET
AN1534.1  	YGGPAYKEWAEAQVQKHKDILNAARADHTNAVKQRMENVKELSGVVDLTKQLFEVSK0ET
          	:*** *.:**: * :* *:***:******:*** *: :*:::..*:::** ** *** **

MG04752.1 	AKLEAEAFELEQKTALAAEAKTVLDSWVRYESQIKQRQQKELAENIIAKVQKELENPKVL
NCU00502.1	AKLEAEAYELEQRTALAAEAKTVLDSWVRYESQVKQRQQKELAQTVIAKVQKELENPKVL
FG00373.1 	AKLESEAFELEQKTALAAEAKAVLESWVRYEGQVKQRQQKELAQSIIAKVQKELENPKVL
AN1534.1  	AKLEAQAYELEQRTALAAEAKQVLDSWVRYEGQVKQRQQRELAETVIGKIQKELQNPKTL
          	****::*:****:******** **:******.*:*****:***:.:*.*:****:***.*

MG04752.1 	QQILQQSIADVEK1IVSSKAQ
NCU00502.1	KQILEQSVADVEK1IVS-KA-
FG00373.1 	QQILQQSVADVEK1IVASKAQ
AN1534.1  	QQILNQSVADVER1IMSSKAQ
          	:***:**:****: *:::**.
```
